# Supplementary figures and images for: Biogenesis of the mitochondrial DNA inheritance machinery in the mitochondrial outer membrane of Trypanosoma brucei
Source: PLoS Pathog. 2017 Dec 29;13(12):e1006808. doi: 10.1371/journal.ppat.1006808 (PMC5764417; doi:10.1371/journal.ppat.1006808)

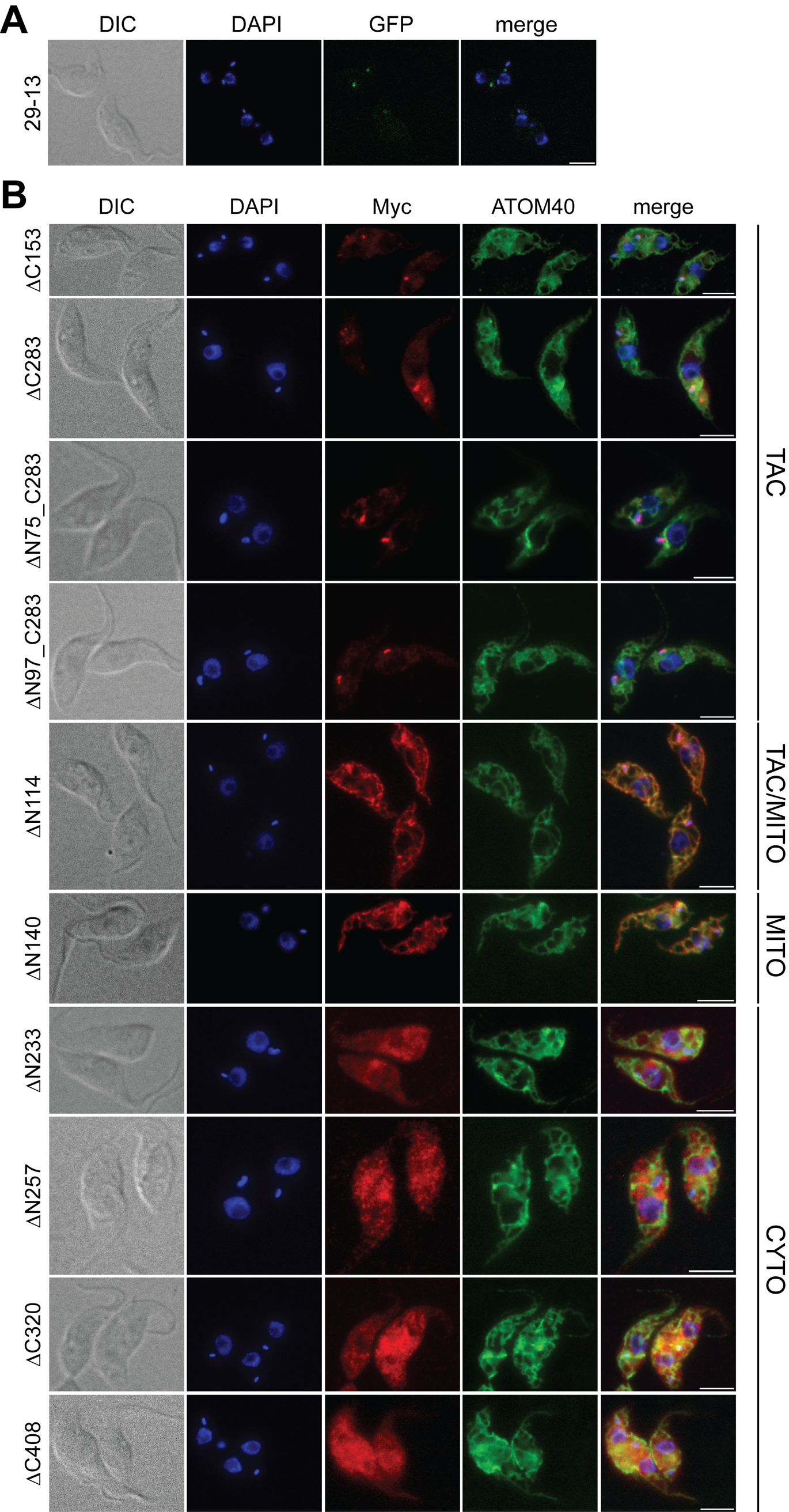

Supplement: S1 Fig — (A) 29–13 cells are analysed for autofluorescence. DIC, differential interference contrast picture. DAPI-stained DNA is shown in blue. Autofluorescence is indicated in green. Bar, 5μm. (B) The cell lines are grouped according to the localization pattern of the TAC60-variants as in shown in Fig 5. DIC, differential interference contrast picture. DNA is stained with DAPI (blue). The Myc-tagged TAC60 variants are indicated in red. The mitochondrial marker ATOM40 is shown in green. The last column shows the merge of all three signals. Co-localization of the TAC60-variants with the kDNA results in a purple signal. Co-localization of the TAC-variants with the mitochondrial marker results in a yellow staining. Bar, 5 μm. (TIF) [file ppat.1006808.s001.tif]

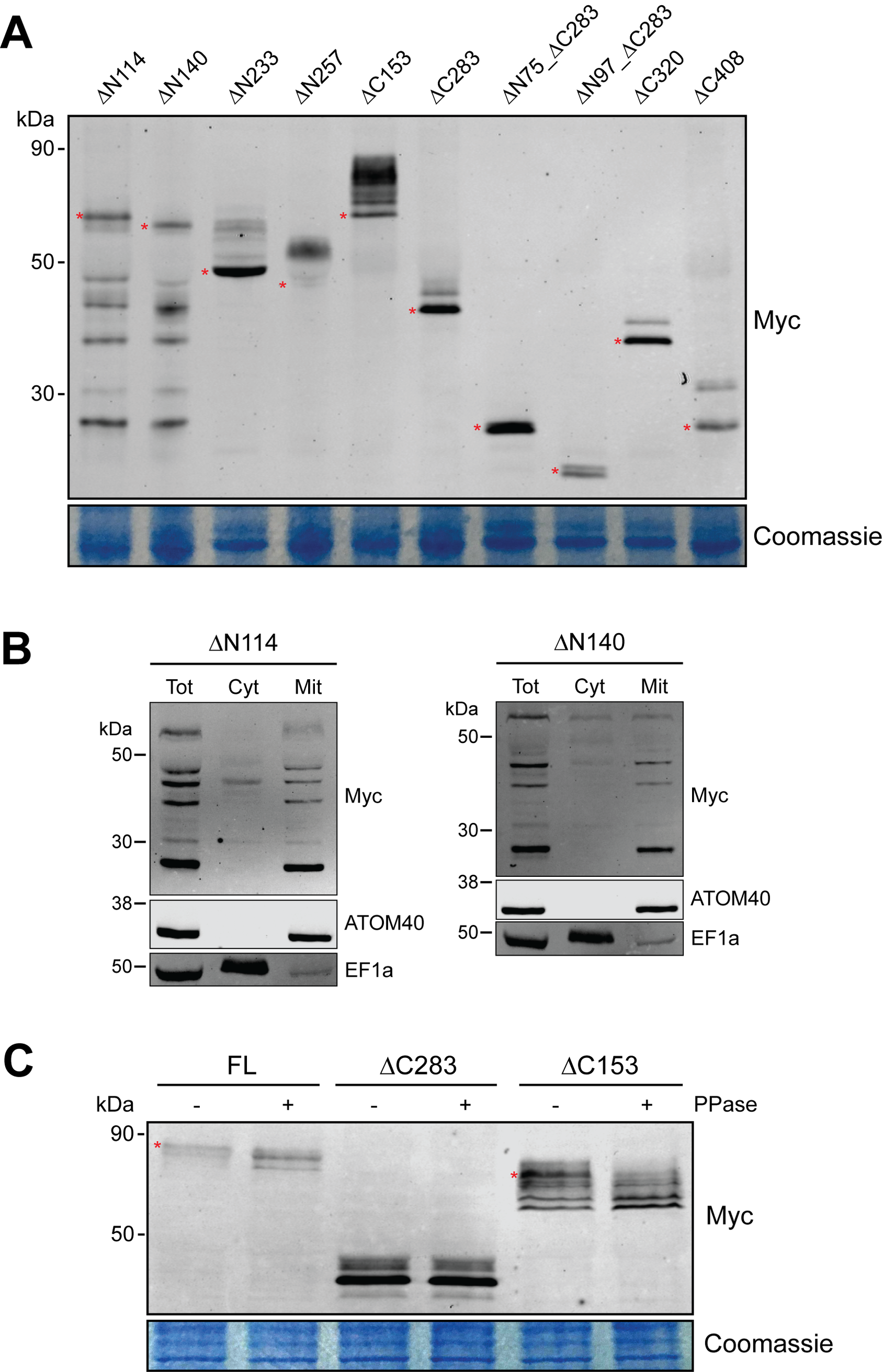

Supplement: S2 Fig — (A) Immunoblot of an SDS-PAGE containing total cellular extracts of the indicated Myc-tagged TAC60-variant expressing cells. Red asterisks indicate which bands of the TAC60-variants most closely match their calculated molecular weight. (B) Immunoblot analysis of whole cells (Tot), soluble (Cyt) and digitonin-extracted mitochondria-enriched pellet (Mit) fractions of cells expressing either the C-terminally Myc-tagged ΔN114 (left panel) or ΔN140 (right panel) TAC60 variant. ATOM40 and EF1a served as mitochondrial and cytosolic markers, respectively. (C) Protein phosphatase (PPase) treatment of total cellular extracts derived from cells expressing the indicated constructs suggests that full length TAC60 and the ΔC153 variant are phosphorylated. Red asterisks indicate which bands are affected by the PPase treatment. The bottom panels in (A) and (C) show a section of the corresponding Coomassie-stained gels that serve as loading controls. (TIF) [file ppat.1006808.s002.tif]
